# Visualizing an unseen enemy; mobilizing structural biology to counter COVID-19^1^

**DOI:** 10.1107/S2053230X20004847

**Published:** 2020-04-06

**Authors:** Edward N. Baker

**Affiliations:** a School of Biological Sciences, University of Auckland, Auckland, New Zealand

**Keywords:** COVID-19, coronaviruses, SARS-CoV-2, editorial

## Abstract

Crystallography in its broadest sense has a crucial role to play in addressing the current COVID-19 pandemic. An outpouring of structural information on key viral proteins has resulted and importantly these data have immediately been shared with researchers round the world to speed the discovery of effective therapeutic agents.

The COVID-19 pandemic currently sweeping round the world reminds us all of our vulnerability to ‘enemies’ we cannot see. In New Zealand we are all in lockdown for the next month, as are many populations, and none of us can see how long this will have to continue. Science of course holds the key to countering the pandemic, but our inability to ‘see’ the agent responsible generates fear, and, in some individuals, disbelief, while it also limits the strategies that can be applied.

Crystallography has a special role to play in this crisis. In its broadest sense, the term crystallography has come to embrace a range of approaches, from traditional single-crystal X-ray analysis through electron and neutron diffraction to cryo-electron microscopy (cryo-EM) and a variety of scattering technologies. Some are relatively mature, while others such as cryo-EM are undergoing revolutionary change. Collectively, they provide a uniquely powerful atomic scale view into the natural world, and when used judiciously, often in combination, can enable the three-dimensional structures of extremely complex molecules and molecular assemblies to be determined with sometimes breathtaking speed.

In many ways the current situation parallels that at the onset of the HIV-AIDS epidemic in the 1980s. In the absence of a straightforward route to a vaccine, crystal structures of key viral proteins, the HIV protease and reverse transcriptase, provided the crucial templates for structure-based drug design, and in a remarkably short space of time effective drugs became available for clinical use. This was arguably the first great triumph for the use of crystallography in drug development. The virus responsible for the present COVID-19 pandemic, known as SARS-CoV-2, is very different from HIV. It belongs to the wider family of coronaviruses that includes those responsible for the SARS (Severe Acute Respiratory Syndrome) and MERS (Middle East Respiratory Syndrome) outbreaks of 2003 and 2012, respectively. These viruses have trimeric protein ‘spikes’ on their surface that have attained an ability to attach to a receptor protein that is widely prevalent on cells of the human respiratory system: the ACE2 receptor. The virus also encodes two long overlapping polyproteins that are processed into smaller, functional, polypeptides that are essential for viral replication. The processing is carried out by two viral proteases, which are now of enormous interest as potential drug design candidates (as was the case for the HIV protease).

What is truly exciting, considering that at time of writing only three months have passed since COVID-19 was first identified, is the speed with which some of these key structures have been determined. Already we have three-dimensional structures for three of them; the Main protease, M^pro^, resolved by crystallography at 2.1 Å resolution by a Chinese group (Jin *et al.*, 2020 ▸); the Spike protein, characterized by cryo-EM by two groups in the USA (Walls *et al.*, 2020 ▸; Wrapp *et al.*, 2020 ▸); and the viral RNA polymerase, characterized by cryo-EM in China (Gao *et al.*, 2020 ▸). The structure of the human ACE2 receptor has also been solved by cryo-EM in China, in complex with the receptor-binding domain of the viral Spike protein (Yan *et al.*, 2020 ▸). There is undoubtedly much more to come as researchers analyse the conformational changes that occur as receptor binding is followed by entry into cells, and target other key viral proteins..

Since its earliest days in the mid-1900s structural biology has been notable for a high degree of collegiality and willingness to share resources, methods and results. This, too, deserves to be celebrated as it is crucial that as many researchers as possible should bring their skills together at a time like this. One manifestation of this collegiality is the role of the worldwide Protein Data Bank (wwPDB), which makes the three-dimensional structural information for macromolecular structures freely available throughout the world. All of the SARS-CoV-2 structures so far determined have been deposited in the wwPDB, checked, annotated and released, most of them in advance of formal publication. This gives researchers anywhere the opportunity to participate in the search for therapies. Already, the M^pro^ structure has been the target of extensive screening in the search for potential drug leads, both by the Chinese researchers themselves and in the United Kingdom at the Diamond synchrotron facility. At the latter site a massive fragment screening exercise is in progress and a web-based call has been made for chemists, drug designers and others to contribute to the initiative.

At this point, research is accelerating and by the time this editorial is published and read, many more advances will have been made. One cannot know what the outcomes of these efforts will be, when a vaccine might become available or whether structure-based drug design will indeed produce effective drugs. My purpose in writing this editorial, however, is to applaud the energy and skill of the researchers, and their public spirit in sharing their results. And I take a vicarious pride in the fact that the discipline to which I have given my professional life – crystallography – has such an important role at a time of international crisis.
